# Newborns of mothers with intellectual disability have a higher risk of perinatal death and being small for gestational age

**DOI:** 10.1111/j.1600-0412.2012.01537.x

**Published:** 2012-12

**Authors:** Berit Höglund, Peter Lindgren, Margareta Larsson

**Affiliations:** Department of Women's and Children's Health, Uppsala UniversityUppsala, Sweden

**Keywords:** Intellectual disability, national registers, newborn outcomes, stillbirth, perinatal death

## Abstract

*Objective.* To study mode of birth, perinatal health and death in children born to mothers with intellectual disability (ID) in Sweden. *Design*. Population-based register study. *Setting.* National registers; the National Patient Register linked to the Medical Birth Register. *Sample.* Children of first-time mothers with ID (*n* = 326; classified in the International Classification of Diseases 8–10) were identified and compared with 340 624 children of first-time mothers without ID or any other psychiatric diagnosis between 1999 and 2007. *Methods.* Population-based data were extracted from the National Patient Register and the Medical Birth Register. *Main outcome measures.* Mode of birth, preterm birth, small for gestational age, Apgar score, stillbirth and perinatal death. *Results.* Children born to mothers with ID were more often stillborn (1.2 vs. 0.3%) or died perinatally (1.8 vs. 0.4%) than children born to mothers without ID. They had a higher proportion of cesarean section birth (24.5 vs. 17.7%) and preterm birth (12.2 vs. 6.1%), were small for gestational age (8.4 vs. 3.1%) and had lower Apgar scores (<7 points at five minutes; 3.7 vs 1.5%) compared with children born to mothers without ID. Logistic regression adjusted for maternal characteristics confirmed an increased risk of small for gestational age (odds ratio 2.25), stillbirth (odds ratio 4.53) and perinatal death (odds ratio 4.25) in children born to mothers with ID. *Conclusions.* Unborn and newborn children of mothers with ID should be considered a risk group, and their mothers may need better individual-based care and support.

**Key Message**

Newborns of mothers with intellectual disability are a risk group. They have a higher risk of being small for gestational age and of stillbirth and perinatal death compared with newborns of mothers without intellectual disability or any other psychiatric diagnosis.

## Introduction

Few studies have described newborn health in children born to mothers with intellectual disability (ID), and knowledge regarding this topic is limited. According to the World Health Organization, ID is defined as an intelligence quotient below 70 and a reduced adaptive capacity that appears before 18 years of age (1). Previous Australian studies found that children born to mothers with ID were more likely than other children to be born preterm (28%), to have low birthweight (22%) and to be admitted to neonatal intensive care (2,3); however, there was no difference in Apgar scores at one minute between children born to mothers with ID and children born to mothers without ID (3). The incidence of preterm birth among all children born in Sweden (1995–2009) was 5% (4) and the incidence of low birthweight (1998–2007) was 6% (5).

The aim of this study was to describe mode of birth, preterm birth rates, Apgar scores, the incidence of being small for gestational age (SGA), stillbirth and overall perinatal death in children born to mothers with ID in comparison to children born to mothers without ID or any other psychiatric diagnosis.

## Material and methods

A total of 326 children born to mothers with ID were compared with 340 624 children born to mothers without ID or any other psychiatric diagnosis between 1999 and 2007 in Sweden. Data were obtained from two of the Swedish National Board of Health and Welfare healthcare registers, namely the National Patient Register (http://www.socialstyrelsen.se/register/halsodataregister/patientregistret/inenglish) and the Medical Birth Register (MBR) (6), which covers 98–99% of all births in Sweden. The variables for comparison were selected on the following grounds: data of good quality (i.e. data with few missing cases) that were found to be important in previous studies (i.e. preterm birth and birthweight; 2,3). The Regional Research Ethics Committee in Uppsala, Sweden, approved this study (327/2007).

A Swedish population-based sample of all women with ID, as defined in International Classification of Diseases (ICD) 8–10, chapter V (ICD-8 codes 311–315, ICD-9 codes 317–319 and ICD-10 codes F70–F79), was identified from the National Patient Register (no women were excluded). The group of women with ID was limited to women with an ID diagnosis and did not include women with syndromes, neuropsychiatric diseases or other psychiatric diagnoses. This sample was linked to the MBR at the National Board of Health and Welfare to identify women with ID who gave birth between 1999 and 2007. A comparison group included all other women who had given birth during the same period, excluding those with any psychiatric diagnosis. Women with ID were younger (mean age 24.2 vs. 28.3 years), with a higher proportion of teenagers (58.6 vs. 22.4%), were more obese (20.1 vs. 8.6%) and smoked more during early pregnancy (27.9 vs. 7.9%) than those without ID. The prevalence of smoking decreased over time in the ID group, from 33.8 (1999–2001) to 33.0 (2002–2004) and then to 19.5% (2005–2007). Other variables included in the analysis were not cohabiting with the child's father at the time of registration (36.6% of mothers with ID vs. 6.2% of those without ID), not working at the time of registration (54.1 vs. 14.3%) and epilepsy (5.5 vs. 0.4%).

Information on single-born children to mothers with ID and to mothers without ID or any other psychiatric diagnosis was obtained from the MBR. The data set extended from 1999 to 2007 and included 505 children born to mothers with ID and 525 664 children born to mothers without ID. To allow a more stringent comparison, only first-born children (those whose mothers were primiparous) were selected, resulting in 326 children born to mothers with ID and 340 624 children born to mothers without ID. The newborn health outcome pattern was the same for both the total sample (505/525 664 and the selected study sample (326/340 624), except for congenital malformations (*p* = 0.038), for which the difference between groups did not remain significant when the sample was reduced to first-borns of primiparous mothers. We chose, however, to analyse the more stringent sample to avoid the influence of parity on the main outcome variables. Preterm birth was defined as a birth before 37 completed weeks of gestation. Small for gestational age was defined as a birthweight and/or birth length of at least two standard deviations below the mean for the infant's gestational age. Stillbirth, according to the Swedish Act before 2008, was defined as an infant born after 28 completed weeks of gestation without signs of life at and after birth. Perinatal death was defined, according to the Swedish convention at the time, as death near the time of birth, from 28 weeks of gestation to one week postnatally. Neonatal death was defined as a child dying in the first 28 days of life. Body mass index (BMI; in kilograms per square meter) was based on height and weight. For the non-pregnant population, a BMI ≤ 24.9 kg/m^2^ was categorized as lean and normal, a BMI of 25.0–29.9 kg/m^2^ overweight and a BMI ≥ 30 kg/m^2^ obese.

### Statistical analysis

Statistical analyses were conducted with the Statistical Package for the Social Sciences (SPSS) 15.0 software program for Windows. Descriptive statistics were used to describe the frequencies of all variables presented. The differences between the case cohort and the control cohort were analysed using the chi-squared test. A significance level of *p* ≤ 0.05 was chosen, and risk ratios with 95% confidence intervals were estimated (Mantel–Haenszel method). Variables that differed significantly between groups in a univariate analysis were inserted into a binary logistic regression analysis. To reveal associations with Apgar score <7 points at five minutes, prematurity, SGA, stillbirth and perinatal death, we used unadjusted binary logistic regression and binary logistic regression adjusted for the effects of maternal characteristics and birth outcome, such as age, BMI, cohabitation with the child's father, working, smoking, epilepsy and cesarean section (CS). Age was used as a continuous variable.

## Results

A greater proportion of children born to mothers with ID than children born to mothers without ID were born by CS (24.5 vs. 17.7%; Table 1). Apgar scores <7 at one and five minutes were more prevalent among children born to mothers with ID than those born to mothers without ID. More children of women with ID compared with women without ID were born preterm (12.2 vs. 6.1%) and were SGA (8.4 vs. 3.1%). Stillbirth was almost four times more prevalent among the children born to mothers with ID than among those born to mothers without ID. Stillbirths were proportionally distributed over time as follows: 2.2% in 1999–2001, 0.9% in 2002–2004 and 0.8% in 2005–2007. Perinatal death was more than four times more common among children born to mothers with ID (1.8%) than among those born to mothers without ID (0.4%).

**Table 1:** Newborn health in children born to mothers without intellectual disability (ID) compared with children born to mothers with ID

	Children of mothers without ID [*n* = 340 624 (%)]		Children of mothers with ID [*n* = 326 (%)]				
					
Characteristic	*n*	Percentage	*n*	Percentage	Relative risk	95% Confidence interval	Missing data (percentage of total data)
Live born	339 551	(99.7)	322	(98.8)	1.0	0.9–1.1	0.0
Cesarean section	60 130	(17.7)	80	(24.5)	1.4	1.1–1.7	0.0
Apgar score							
One minute <7 points	21 634	(6.4)	32	(9.8)	1.5	1.1–2.2	0.7
Five minutes <7 points	5064	(1.5)	12	(3.7)	2.5	1.4–4.4	0.8
Ten minutes <7 points	1967	(0.6)	3	(0.9)	1.6	0.5–4.9	3.7
Born preterm							0.1
Weeks 22–29	1712	(0.5)	5	(1.5)	3.0	1.3–7.3	
Weeks 30–36	19 055	(5.6)	35	(10.7)	1.9	1.4–2.7	
Weeks 37–42	317 022	(93.1)	284	(87.1)	0.9	0.8–1.1	
Weeks 43–45	2483	(0.7)	2	(0.6)	0.8	0.2–3.4	
Small for gestational age	10 466	(3.1)	27	(8.4)	2.7	1.9–3.9	0.7
Large for gestational age	7203	(2.1)	7	(2.2)	1.0	0.5–2.1	0.7
Congenital malformation	12 878	(3.8)	18	(5.5)	1.5	0.9–2.3	0.0
Stillbirth	1073	(0.3)	4	(1.2)	3.9	1.5–10.4	0.0
Perinatal death	1455	(0.4)	6	(1.8)	4.3	1.9–9.6	0.0
Neonatal death	525	(0.2)	2	(0.6)	4.0	1.0–16.0	0.0

The multivariate analysis included the outcome variables that differed between the groups in the univariate analysis (Table 2). In the logistic regression analysis, an ID diagnosis was associated with Apgar score <7 at five minutes, preterm birth, SGA, stillbirth and perinatal death (crude odds ratio). When the model was adjusted for effects of maternal characteristics and mode of birth, the ID diagnosis was associated with SGA, stillbirth and perinatal death (adjusted odds ratio). In a subanalysis, we compared smoking mothers with ID vs. non-smoking mothers with ID, and the result did not differ between the groups with respect to Apgar score <7 points at five minutes, prematurity, SGA and perinatal death.

**Table 2:** Crude odds ratio (95% confidence interval) and adjusted odds ratio (95% confidence interval) for preterm birth, Apgar score <7 points at five minutes, small for gestational age, perinatal death and stillbirth in women with intellectual disability (ID) compared with women without ID

	Women with ID vs. women without ID			
	
Characteristic	Crude odds ratio	(95% confidence interval)	Adjusted odds ratio	(95% confidence interval)
Preterm birth	2.15	(1.55–3.00)	1.58	(0.99–2.51)
Apgar <7 points at five minutes	2.59	(1.45–4.61)	1.83	(0.81–4.15)
Small for gestational age	2.86	(1.93–4.24)	2.25	(1.37–3.69)
Perinatal death	4.37	(1.95–9.19)	4.25	(1.72–10.50)
Stillbirth	3.93	(1.46–10.56)	4.53	(1.65–12.44)

Odds ratios were adjusted for maternal age, obesity, cohabitation with the child's father, working, smoking, epilepsy and cesarean section.

## Discussion

The main findings of this study included an increased risk for mothers with ID to have children being SGA, and an overrepresentation of stillbirths and perinatal deaths among children born to mothers with ID. A higher proportion of children of mothers with ID were born preterm and by CS.

An Australian study revealed a higher incidence of pre-eclampsia in women with ID (3), and pre-eclampsia is associated with SGA (7). We did not have information about pre-eclampsia among the mothers of children in this study, but it cannot be exluded that this higher incidence of pre-eclampsia also exists in Sweden. Stillbirths and perinatal death among children born to intellectually disabled mothers were four times higher than the proportion among children born to mothers without ID; 1.2 vs. 0.3 and 1.8 vs. 0.4%, respectively. Increasing maternal age, obesity, unemployment and smoking were also associated with stillbirth, peri- and neonatal death, which is supported by several studies (8–15). The relative reduction in smoking over time among mothers with ID did, however, not significantly change the proportion of stillbirths in our study. Decreased fetal movements commonly precede stillbirth, but we still lack proper evidence on how to use fetal movement assessments in clinical practice to prevent stillbirths (16). Mothers with ID may have greater difficulties than mothers without ID in understanding and reacting to decreases in fetal movements.

A decrease in fetal movements is likewise a well-known risk factor for SGA, and in our study children born to mothers with ID were more often SGA (8.4%) than those born to mothers without an ID (3.1%). We also found an association between SGA and older maternal age, not living with the child's father, unemployment, CS and smoking. Prenatal smoking has previously been reported to increase the risk of SGA (odds ratio 3.08) (17). Maternal smoking at the first antenatal visit has decreased over time in Sweden from 31 (1983) to 7% (2008) (18). This development probably reflects an increased understanding among the general public of the health risks associated with smoking during pregnancy. Despite the decrease in smoking over time, there was an overrepresentation of smoking among mothers with ID compared with mothers without ID. This could partly be explained by their younger mean age at childbirth. However, other reasons (i.e. inadequate information about smoking, difficulty in understanding the information, a desire to keep smoking or inadequate support for smoking cessation) cannot be excluded.

In the present study, there were no data about alcohol or substance abuse other than tobacco among the mothers. It is likely that women with ID live in more deprived settings, which could lead to alcohol and/or substance misuse. Moderate to severe predelivery depression, anxiety and stress among mothers with ID were inferred in an Australian study (19), and maternal substance use was a risk factor for SGA (odds ratio 2.40) (20). Such information was not available from the register data that we used, but it can be assumed that if mothers with ID more often use benzodiazepines during pregnancy, the use of such drugs could contribute to the greater incidence of preterm births, SGA and lower Apgar scores in their children compared with the children of mothers without ID. The maternal use of benzodiazepines during pregnancy is believed to increase the risk of preterm delivery (adjusted odds ratio 6.79), low birthweight, low Apgar score, neonatal intensive care, respiratory distress syndrome (21) and CS (22,23). There are conflicting results regarding selective serotonin reuptake inhibitor treatments during pregnancy. A Canadian study affirmed that maternal use of selective serotonin reuptake inhibitor antidepressants is associated with preterm birth, low birthweight and respiratory distress (24). Furthermore, a recent study has documented an increased risk of persistent pulmonary hypertension of the newborn (25). However, a recent review article claims that the suspected increase in risk of preterm birth, low birthweight or SGA has not been confirmed (26).

Apgar score at one minute was not shown to differ between children born to women with ID compared with those born to women without ID (3). Our study suggested a lower Apgar score at five minutes in women with ID compared with women without ID, but the lower Apgar score was probably due to maternal characteristics and mode of delivery. In the multivariate analysis, after adjusting for these variables we were unable to verify an association between Apgar score <7 at five minutes and ID. A higher proportion (12.2%) of children born to mothers with ID were born preterm, compared with children born to mothers without ID (6.1%). Preterm birth was also associated with maternal obesity, smoking and CS. Prenatal smoking has in previous studies demonstrated an increased risk of preterm delivery (27), as well as extreme prematurity (odds ratio 7.25) (17). The increased preterm birth rate in women with ID could be compared with results from Australian studies with 28% preterm births (2) and an odds ratio of 1.76 (99% confidence interval 0.59–5.28) compared with a reference group of women without ID (3). The increased risk of preterm birth in women with ID is most probably due to CS and/or other maternal characteristics than to their ID diagnosis, because no association was found in the logistic regression analysis after adjusting for these variables.

The maternal characteristics and the mode of birth in children born to women with ID increase the risk for low Apgar score and preterm birth. Together with an increased risk of low birthweight, it is more likely that there will be complications for the infant and more frequent admissions to a neonatal intensive care unit with such issues as dyspnea, temperature drops, infections, cramps, and difficulty with breastfeeding and bonding with the mother. A recent study from the UK (28) stated that it is more common for children born to mothers with mild to moderate ID to be admitted to a special neonatal care unit and not to be breastfed at discharge.

In general, antenatal care attendance in Sweden is very high. Roughly 90% of pregnant women have made an initial antenatal visit within the first 12 full pregnancy weeks (6). A strength of the present study is the prospective nature; recall bias was avoided by using data gathered at maternal care units and hospitals. In Sweden, maternal care is free of charge and home deliveries are rare; thus, selection bias is unlikely. One weakness of the MBR is its lack of certain documentation, such as missing records, incorrect documentation, changes in medical records, and incorrect transmission into the MBR (6). It is a shortcoming that medical records do not include data on education level. If this variable were available, it could serve as a proxy for socioeconomic status in the MBR. The clinical significance of Apgar score at one minute is weak, and Apgar score registration at 10 minutes is often missing because midwives commonly do not register the Apgar score at 10 minutes if it has reached 10 points at one and/or five minutes. We therefore concentrated the analysis on Apgar score at five minutes. The place to which the children were discharged (i.e. directly to home or to another place) could have been a useful variable for analysis in this study; however, there were too many missing values for child discharge. Data on discharge are registered in separate medical records, one for the mother and one for the child, and the transfer of data to MBR from these records may occur while the child is still in the neonatal unit. The congenital malformation variable included various deformations and chromosomal abnormalities, but an analysis of the subgroups within this variable was not possible.

The women with ID were identified in the National Patient Register, which means that they have a registered facility care or out-patient care on some occasion. They are therefore a clearly defined sample, but there may be other mothers with ID not registered in the National Patient Register, and we cannot claim that our sample is representative for all women with ID in Sweden, because it probably consists predominantly of women with mild ID. We had no data about the underlying causes of ID or its degree of severity. It has, however, been reported that women with a mild ID more often have children than women with a moderate or severe ID (29). We have no reasons to believe that this sample represents the most severe cases of ID and that differences between groups are overestimated. We had no data about lifestyle factors (except smoking) and use of medical products or the mothers’ socioeconomic situation, all of which may have an impact on pregnancy outcomes. The results of this study cannot be generalized to other populations, although it is reasonable to believe that the same findings would occur in populations with sociodemographic conditions similar to those of the Swedish population.

In conclusion, unborn and newborn children of mothers with ID have a higher likelihood of perinatal death or being SGA. The children are more often born preterm and by CS. These pregnant women and their newborns should therefore be considered a risk group. Furthermore, we believe that there is a need to increase the knowledge and skill of health professionals caring for mothers with ID to enable healthy choices during pregnancy for these women, to enhance their preparedness for birth and their ability to care for the newborn child.

## Abbreviations

BMI: body mass index
CS: cesarean section
ICD: International Classification of Diseases
ID: intellectual disability
MBR: Medical Birth Register
SGA: small for gestational age
